# Medical management of vesicoureteral reflux

**DOI:** 10.1007/s00467-007-0485-3

**Published:** 2007-08-01

**Authors:** Tej K. Mattoo

**Affiliations:** Division of Pediatric Nephrology, Children’s Hospital of Michigan, Detroit, MI USA

**Keywords:** Vesicoureteral reflux, Treatment, VUR

## Abstract

Vesicoureteral reflux (VUR) in children is associated with increased risk of urinary tract infection (UTI). Recurrent UTI in the presence of the VUR is believed to cause renal scarring, which carries a risk of subsequent hypertension, toxemia of pregnancy, and significant renal damage, including end-stage renal disease. The natural history of VUR is to improve or resolve completely with time in most of the patients. The traditional management consists of prompt treatment of UTI, long-term anti-microbial prophylaxis until the VUR resolves, or surgical intervention in those with persistent high grade VUR, recurrent UTI in spite of prophylaxis with anti-microbial agent, allergy to anti-microbial agents, and patient/parent non-compliance with the medical management. Voiding dysfunction and constipation play an important role, and their diagnosis and appropriate management helps reduce the frequency of UTI and promote the resolution of the VUR. Patients with renal scarring need to be monitored for potential complications such as hypertension, proteinuria, and progression of the renal damage. In patients with hypertension and/or proteinuria, angiotensin-converting enzyme inhibitors (ACEIs) or angiotensin II receptor blockers (ARBs) are the drugs of choice, because of their reno-protective properties. Recent studies have revealed that there is no convincing evidence that UTI in the presence of VUR predicts renal injury or that the use of long-term anti-microbial prophylaxis or surgical intervention prevents renal scarring or its progression. However, until proven otherwise by a prospective, placebo-controlled, randomized study, it is advisable to err on the side of caution and consider VUR and UTI risk factors for renal scarring and treat each patient on individual basis.

## Introduction

Primary vesicoureteric reflux (VUR) is the most common urological abnormality in children, with a prevalence of approximately 1% [1]. It may be an isolated finding, or it may occur in association with other anatomic renal abnormalities, such as multi-cystic dysplastic kidney, renal agenesis, or ureteral ectopia. In a significant number of cases, it is diagnosed after urinary tract infection (UTI). According to the various reports, it is present in 8%–50% of children, predominantly girls, with febrile UTI [2–4]. Many newborn babies are diagnosed with VUR during follow-up for antenatally diagnosed hydronephrosis (antenatal VUR). Approximately 10% of fetuses with renal pelvic diameter of greater than 5 mm at or beyond 28 weeks of gestation are found to have VUR during examination after birth [5]. Antenatal VUR, which is more common in boys, is usually low grade, and the possibility of resolution is high [5, 6]. Some cases of VUR are also diagnosed during family screening of a known sibling or a parent with VUR; the incidence of VUR in such cases is approximately 30% [7].

VUR predisposes to UTI, and the two are associated with renal scarring (reflux nephropathy), which carries a risk of subsequent hypertension, toxemia of pregnancy, and significant renal damage, including end-stage renal disease. According to the North American Pediatric Renal Transplant Cooperative Study (NAPRTCS) annual report of 2006, 536 (8.4%) of the 6,405 children with chronic renal insufficiency had reflux nephropathy, which, according to the registry, is the fourth commonest cause of chronic renal insufficiency after obstructive uropathy, renal aplasia/hypoplasia/dysplasia, and focal segmental glomerulosclerosis (FSGS) [8].

The natural course of VUR is spontaneous resolution in 25% to 80% of cases, and it depends on the severity of VUR and the duration of follow-up. Resolution may be delayed by recurrent UTI, voiding dysfunction, and chronic constipation [9, 10].Various treatment strategies have been used in children with VUR, with the ultimate objective of preventing renal injury. The two main treatment modalities are long-term anti-microbial prophylaxis and surgical correction. Since the natural tendency of the VUR is to improve with time, medical management is generally tailored to cover the period during which the VUR is likely to cause the renal damage.

## Natural history of VUR

The Birmingham Reflux Study, which interpreted negative findings in a single voiding cysto-urethrogram (VCUG) as the resolution of VUR, reported an approximately 50% cessation of VUR after a 5-year follow up in medically managed moderate-to-severe VUR [11]. In the International Reflux Study in Children, which used the negative findings of two consecutive VCUG examinations to confirm resolution of VUR, the corresponding number was 25% [12]. The rate of resolution is better in undilated ureters than in dilated ureters, and in low-grade VUR than in high-grade VUR. The International Reflux Study in Children reported that VUR disappeared in more than 80% of undilated ureters and in about 40% of dilated ureters [2]. Schwab et al. reported that grades I–III VUR resolved at the rate of 13% per year for the first 5 years of follow-up and 3.5% per year during subsequent years. Grades IV and V VUR resolved at the rate of 5% per year [13]. Some studies have also reported that the cessation of VUR occurs sooner in children with unilateral VUR than in those with bilateral VUR [12, 14].

Resolution of VUR occurs sooner in African–Americans. In a study on black children, the mean duration to spontaneous resolution was 14.6 months, which was significantly shorter than the 21.4 months in white children. No significant difference was seen in the rate of spontaneous resolution [15]. In newborn babies with VUR diagnosed after abnormal findings in prenatal renal ultrasound (US) (prenatal VUR), 67% of severe VUR and 78% of mild or moderate VUR had resolved by the age of 2 years [16].

After a systematic review of published literature on the resolution of VUR, Elder and colleagues reported that increasing age at presentation and the presence of bilateral VUR decrease probability of resolution and that bilateral grade IV VUR has a particularly low chance of spontaneous resolution [17].

## Medical management versus surgical management

Surgical correction of VUR was common until the concept of anti-microbial prophylaxis for childhood UTI was introduced in 1975 [18]. Numerous subsequent studies revealed that medical management of VUR is as effective as surgical treatment and that there is no significant outcome difference between the two treatment modalities. In the International Reflux Study Group in Europe, 287 children with severe VUR were randomly allocated to medical (*n* = 147) or surgical (*n* = 140) groups. Follow-up with di-mercaptosuccinic acid (DMSA) renal scans for a period of 5 years revealed no difference in outcome between children managed surgically or medically [19].

In the International Reflux Study in Children (IRSC), which included 306 patients, no significant difference in outcome was found between medical or surgical management in terms of the development of new renal lesions or the progression of established renal scars [20]. Similar results were reported by the Birmingham Study [11]. In its final report, the IRSC compared long-term outcome of medical management versus surgical management in 252 children less than 11 years old with non-obstructed grade III/IV VUR, history of UTI, and a glomerular filtration rate (GFR) of >70 ml/min per 1.73 m^2^ body surface area. The follow-up period was 10 years. UTI recurrences and renal growth were similar in the two groups, but the medically treated group had more febrile infections. The report concluded that, with close supervision and prompt treatment of UTI recurrence, children in medical or surgical groups did equally well [21].

Medical management seems appropriate for VUR grades I–III unless the patient has recurrent breakthrough UTI, is allergic to the anti-microbial agents, or has compliance issues. For VUR grade IV, the choice of medical management versus surgical management is controversial. The spontaneous resolution rate is <40% after 5-year follow up. Decision for surgical intervention depends on the patient’s age, renal function, duration of follow-up, and other factors as for those with VUR grades I–III. VUR grade V has the lowest rate of spontaneous resolution, particularly in older children, and some advise surgical intervention if there is no improvement within a year—certainly if the patient has recurrent infections while on anti-microbial prophylaxis [12, 22, 23].

## Anti-microbial prophylaxis

The medical management of VUR consists primarily of long-term anti-microbial prophylaxis. This approach is endorsed by many professional societies, which include the American Academy of Pediatrics [24], the Swedish Medical Research Council [25], and the American Urological Association [17]. Anti-microbial agents most appropriate for prophylaxis include trimethoprim-sulfamethoxazole (TMP-SMZ), trimethoprim alone, nitrofurantoin, or cephalexin [24, 26]. According to one report, nitrofurantoin is more effective than TMP-SMZ as a prophylactic agent [27]. In view of an increasing resistance of *E. coli*, ampicillin and amoxicillin are less effective as prophylactic agents [24, 28, 29] and are not used for this purpose beyond the first 2 months of the child’s life, during which period it is advisable to avoid TMP-SMZ. In toilet-trained children, the medication is generally administered at bed time, though this recommendation is not evidence based.

The prophylactic dose of anti-microbials is one-fourth to one-half of the therapeutic dose for acute infection. The dosage for commonly used anti-microbials is as follows [24, 26]:

**Table:** 

TMP-SMZ	TMP 2 mg per kg as a single dose, or
	5 mg of TMP per kg twice per week
Nitrofurantoin	1–2 mg/kg as a single daily dose
Cephalexin	10 mg/kg as a single daily dose
Ampicillin	20 mg/kg as a single daily dose
Amoxicillin	10 mg/kg, as a single daily dose

In 1997, the American Urologic Association (AUA) published its guidelines on the management of VUR in children. The recommendations were based on a systematic review that involved 168 articles on VUR that were published from 1965 to 1994. The seven treatment modalities that were studied included (1) intermittent antibiotic therapy, (2) bladder training, (3) continuous antibiotic prophylaxis, (4) antibiotic prophylaxis and bladder training, (5) antibiotic prophylaxis, anti-cholinergics and bladder training, (6) open surgical repair, (7) endoscopic repair. The key outcome measures were: resolution of VUR, risk of pyelonephritis and scarring, and complications of medical management versus surgical management. The study panel recommended antibiotic prophylaxis for all grades of VUR in children less than a year of age because of a very high rate of spontaneous resolution. For children 1–5 years old, antibiotic prophylaxis is recommended for all grades of VUR, with surgical options in grades III to V if VUR is bilateral or renal scarring is present. For children older than 6 years antibiotic prophylaxis is recommended for grades I and II (unilateral or bilateral) and unilateral grades III and IV, with surgical options if renal scarring is present; and surgical repair for bilateral grade III and IV, and unilateral or bilateral grade V VUR with or without scarring since the VUR has the least possibility of spontaneous resolution [17].

The duration of anti-microbial prophylaxis and the potential need for surgical intervention depends on the age of the patient, the severity of the VUR, frequency of UTI, and the degree of renal scarring, if present. Some recommend cessation of anti-microbial prophylaxis after the ages of 5–7 years, even if low-grade VUR persists [30]. The Swedish Medical Research Council published its recommendations in 1999, which recommended no anti-microbial prophylaxis for VUR grades I and II at first examination and a follow up with DMSA renal scan at one year. For those with VUR grades III and IV on first examination, anti-microbial prophylaxis for 1 year is recommended, at which time it should be discontinued if the VUR grade has decreased to grades 0–II. If there is no change in the VUR grade, and the patient is a boy, the prophylaxis can be discontinued, whereas its continuation of prophylaxis or surgical intervention is recommended for such girls. For grade V VUR at all ages and bilateral grade IV in children less than a year old on the first examination, the panel recommended that anti-microbial prophylaxis should be started, the management should be individualized, and surgical intervention may be considered if there is no change in VUR after 1 year. The importance of bladder function evaluation and hygiene, and a careful follow-up of patients, including DMSA renal scan, was emphasized [25].

Follow-up of patients with VUR and UTI requires close monitoring for the early detection and prompt treatment of UTI, changing of anti-microbial prophylaxis if the patient has recurrent breakthrough UTI , and the monitoring of the VUR by periodic VCUG examinations. The timing for follow-up VCUG is not well-defined, though most practitioners do it yearly. In an effort to estimate the optimum timing for follow-up VCUG examinations, Thompson et al. derived a decision-tree model from the published data on VUR and validated the model by using a retrospective cohort of 76 patients. The authors concluded that delaying the VCUG from yearly to every 2 years in children with mild VUR (grades I and II) and every 3 years in children with moderate/severe VUR (grades III or higher) yields substantial reductions in the average numbers of VCUG examinations and costs, with a modest increase in anti-microbial exposure [31].

### Limitations of anti-microbial prophylaxis

Long-term anti-microbial prophylaxis has its limitations. It is not always effective: the breakthrough UTI rates in children with VUR range from 25%–38% [11, 12]. Anti-microbial resistance is a major concern with long-term anti-microbial prophylaxis. In one study, children who had received the medication for more than 4 weeks in the preceding 6 months had more resistant *E. coli* than did those not on such treatment [odds ratio (OR) = 13.9; 95% confidence interval (CI) 8.2–23.5] [32]. In another study on childhood UTI, a generalized decrease in bacterial susceptibility to common antibiotics was seen in 1999 when compared with those previously seen in 1991 [32]. Approximately 10% of children on long-term prophylaxis have adverse reactions, most of which occur within the first 6 months. These include gastrointestinal symptoms, skin rashes, hepatotoxicity, and hematological complications with SMZ-TMP, and mostly gastrointestinal symptoms with nitrofurantoin. More severe adverse reactions, such as marrow suppression, and, rarely, Stevens–Johnson syndrome, may also occur with SMZ-TMP [33, 34]. Compliance with daily administration of the medication over a prolonged period of time is questionable. In one study, 97% of the parents reported compliance with low-dose daily anti-microbial prophylaxis, and yet the medicine was found in only 31% of the patients’ urine [35]. Other concerns with long-term anti-microbial prophylaxis are the inconvenience to patients with repeated follow-up VCUG examinations to monitor the VUR resolution, and the cost of the procedure.

### Controversies over anti-microbial prophylaxis

Many studies have raised serious doubts about the efficacy of long-term anti-microbial prophylaxis in the prevention of renal injury in patients with VUR. Shindo et al. observed that renal scarring, the injury presumably having been initiated by VUR, can progress despite correction of the reflux and prevention of UTI [36]. Arant et al. reported that, in spite of good medical management, even mild and moderate VUR can be associated with renal injury [37]. In a study on 51 children (mean age 8.6 years) with VUR (grades I–IV), the prophylactic antibiotic was discontinued after a mean period of 4.8 years. After a mean follow-up period of 3.7 years, only 11.8% patients had UTI, and no new scars had developed in any of the patients [30]. Other reports that raise doubts about the usefulness of long-term anti-microbial prophylaxis include the observation that up to half of patents with severe VUR exhibit no evidence of renal damage [38], incidence of renal scars does not always match the severity of VUR [39], and the frequency of pyelonephritis is similar with or without the resolution of VUR [12]. In a randomized study involving 113 children with grade I–III VUR in the age group of 3 months to 12 years, Garin et al. reported no difference in recurrence rate of UTI and renal scarring at 1 year between those who received prophylaxis and those who did not [40].

In a systematic analysis that compared antibiotics with placebo or no treatment for preventing UTI in susceptible children, Williams and colleagues concluded that most published studies to date have been poorly designed, with biases known to overestimate the true treatment effect [41]. Another systematic analysis, which evaluated the value of identification of VUR after symptomatic UTI and the effects of various interventions on the occurrence of UTI and subsequent renal parenchymal damage, concluded that it is uncertain whether the identification and treatment of children with VUR confers clinically important benefit and whether any intervention, including antibiotic prophylaxis or surgery for VUR, is better than no treatment [42].

In view of a lack of consensus over the management of VUR and increasing doubts about the relevance of long-term anti-microbial prophylaxis in preventing renal damage, the National Institutes of Health (NIH)/National Institute of Diabetes and Digestive and Kidney Diseases (NIDDK) has initiated a multicenter, prospective, randomized, placebo-controlled study that will investigate the role of anti-microbial prophylaxis in preventing recurrent UTI and renal scarring in patients with VUR. This study will include children aged 2 months to 5 years with grades I–IV VUR with first UTI.

## Voiding dysfunction and VUR

VUR may be associated with voiding dysfunction, which, in some cases, may present as dysfunctional elimination syndrome (DES). The symptoms of DES include daytime wetness, urgency, frequency, infrequency, constipation or fecal incontinence in “toilet trained” children, with no underlying anatomic or neurological abnormality [43–45]. The exact pathogenesis of voiding dysfunction or DES is not known. It seems to be an abnormally learned spectrum of voiding, which evolves from poorly learned voiding technique and attempts to suppress impending or active bladder contractions by inappropriately contracting the pelvic floor muscles and tightening the sphincter [44, 46]. This results in increased voiding pressure and/or inefficient voiding [47, 48].

Voiding dysfunction predisposes to recurrent UTI, induces and perpetuates VUR, and may result in permanent renal damage [9, 10]. In a study by Koff et al. DES, besides increasing the rate of breakthrough UTI, it delayed resolution of VUR that was 1 grade less severe by an average of 1.6 years. DES also adversely affected the results of ureteric re-implantation [45]. In another study that involved the use of a questionnaire in 310 children enrolled in the European branch of the International Reflux Study in Children, a strong negative correlation was seen between recurrences of urinary tract infections, as well as disappearance of VUR and non-neuropathic bladder/sphincter dysfunction [49].

Voiding dysfunction or DES is a diagnosis of exclusion that mandates the taking of a detailed history and a physical examination to rule out any underlying neurological or neuromuscular etiology. The diagnosis is usually evident clinically, and urinalysis, bladder ultrasound, and VCUG in selected cases are helpful in making a diagnosis. The role of urodynamic studies is not well established, partly because the study result is not consistent [44, 50, 51]. The procedure is invasive, and the study result does not change therapy or influence the final outcome. A thorough history taking and physical examination lead to the correct diagnosis and treatment in the majority of children [52].

The treatment of voiding dysfunction or DES may include the use of laxatives and timed frequent voiding every 2 to 3 hours. Pelvic floor exercises, behavioral modification, and/or anti-cholinergic medication may be required. A combination of conservative medical and computer game-assisted pelvic floor muscle retraining decreased the incidence of breakthrough UTI and facilitated VUR resolution in children with voiding dysfunction and VUR [53]. Similar results of improved outcome with medical management have been reported by others [54, 55].

## Constipation and VUR

Besides being a part of dysfunctional elimination syndrome, constipation may be an isolated finding, which by itself increases the risk of recurrent UTI or voiding dysfunction in children with VUR. This is believed to result from compression of the bladder and bladder neck that increases bladder storage pressure and post-void residual urine volume. Also, a distended colon and/or soiling provide an abundant reservoir of pathogens [56–58]. Constipation in children increases the likelihood of urinary incontinence, bladder overactivity, discoordinate voiding, a large capacity and poorly emptying bladder, recurrent UTI, and deterioration of VUR [59]. In a study that involved 366 children, constipation/encopresis was reported in 30% of cases, day-time wetting in 89%, night wetting in 79%, and recurrent UTI in 60% of the patients. VUR was present in 20% of the patients who underwent cysto-urethrography. Multi-disciplinary management helped the resolution of VUR in 53% of the patients, after a mean follow-up period of 22 months [55].

Treatment of constipation by dietary measures, behavioral therapy, and laxatives helps reduce UTI recurrence and the resolution of enuresis and uninhibited bladder contractions [57, 60].

## VUR and renal scarring

Many children with VUR have renal scars at the time of diagnosis or develop new scars during the follow-up. In a study involving 127 adults (mean age 41 years) with VUR diagnosed during childhood, 44 (35%) had unilateral renal scarring, 30 (24%) had bilateral renal scarring, 12 (9%) had proteinuria, 30 (24%) had albuminuria, and 14 (11%) patients had hypertension. Of the 30 patients with bilateral renal scars, 25 (83%) had an abnormal GFR [61]. Another study on 21 adults (mean age 23.9 years, range 16–37 years) with gross VUR diagnosed in infancy revealed that three (23%) of the 13 patients with unilateral reflux nephropathy and two (50%) of the four patients with bilateral reflux nephropathy had proteinuria and renal insufficiency [62]. A high incidence of micro-albuminuria has also been reported in children with pyelonephritic renal scarring. A study involving 57 such children (1–17 years old) revealed significantly lower GFR (median 93 ml/min per 1.73 m^2^ vs 111 ml/min per 1.73 m^2^ in controls) and higher urine albumin excretion (median 20 μg/min per 100 ml GFR vs 9.2 μg/min per 100 ml GFR in controls). An inverse correlation was found between urine albumin excretion and GFR. Increased urine albumin excretion (>20 μg/min per 100 ml GFR) was found in 51% of the children with pyelonephritic scarring; only 14% had increased serum creatinine [63].

The predisposing factors for renal scarring include younger age, presence of urological abnormality, and recurrent UTI [2, 64, 65]. The follow-up of children with renal scarring, with or without the resolution of VUR, is important because of a higher risk of hypertension, proteinuria, and the progression of the renal disease [62, 63, 66, 67]. These complications are attributed to hyperfiltration in the remnant glomeruli following renal parenchymal loss, which leads to the development of glomerulosclerosis, activation of the renin–angiotensin system, and a gradual deterioration of renal function [68, 69] Studies have indicated that the mesangial alterations occur early in the course of reflux nephropathy, even before proteinuria is detectable by routine analysis; the other extreme is the occurrence of focal segmental glomerulosclerosis (FSGS) with clinically significant proteinuria and nephrotic syndrome [70].

The exact cause of hypertension due to renal scarring is not known, but it is believed to be due to segmental ischemia with increased renin secretion, and it does not depend on the severity of the scarring [71–73]. Of the 306 patients entered into the IRSC, three had hypertension at study entry. Of the three, two became normotensive within 5 years of follow-up and the third one required anti-hypertensive medication at the end of the study. Three more patients became hypertensive during follow-up [21]. In another study involving 664 patients diagnosed with VUR between 1970 and 2004, 20 (3%) developed hypertension. The estimated probability of hypertension was 2% (95%CI 0.5–3%), 6% (95%CI 2–10%), 15% (95%CI 11–20%) at 10 years, 15 years, and 21 years of age, respectively. The survival analysis revealed that approximately 50% of children with unilateral and bilateral renal damage had developed persistent hypertension at about 30 years and 22 years of age, respectively. The presence of hypertension strongly correlated with the renal damage at entry [67].

Appropriate management of hypertension and/or proteinuria is important to slow down the progression of the renal disease. Even though there is a large number of available anti-hypertensive drugs, the angiotensin-converting enzyme inhibitors (ACEIs) or angiotensin II receptor blockers (ARBs) are the preferred ones because of their renoprotective effect. Studies have revealed that ACEIs, in addition to lowering the blood pressure, reduce proteinuria due to reflux nephropathy [74] A combination of ACEIs and ARBs significantly improves the renoprotective effects of ACEIs [75]. However, it is not known whether this anti-proteinuric effect slows down the progression of the renal disease. In some patients with one very poorly functioning scarred kidney and a healthy other kidney, the removal of a poorly functioning kidney may help cure hypertension [76].

## Conclusion

Serious doubts exist about the role of long-term anti-microbial prophylaxis in preventing renal injury in children with VUR. This is because the current recommendations are based on non-randomized studies that were not placebo controlled and that included patients with pre-existing renal damage that was diagnosed after the onset of UTI. Systematic reviews of the published literature on the subject have highlighted these shortcomings. However, until the results of an appropriately designed, placebo-controlled, prospective study become available, the prudent thing to do is to assume that VUR is a risk factor for renal scarring and treat each patient on an individual basis, with due attention being paid to voiding dysfunction, constipation, and renal scarring, if present.

## Multiple choice questions (Answers appear following the references)

1. Which ONE of the following is the appropriate anti-hypertensive medication in a 9-year-old boy with right reflux nephropathy and mild proteinuria with normal blood chemistry?
  A. Angiotensin-converting enzyme inhibitor
  B. Calcium channel blocker
  C. Hydrochlorothiazide
  D. Beta blocker
2. Which ONE of the following is true of anti-microbial prophylaxis in children with VUR?
  A. The long-term renal outcome is better than that of surgical intervention
  B. Amoxicillin is the drug of choice in a 2-year-old patient
  C. There is no convincing evidence that it is better than a placebo in preventing renal injury
  D. About 10% of patients have adverse reactions
3. Which ONE of the following is true of renal injury in VUR?
  A. The incidence of renal scars always matches the severity of VUR
  B. Correction of VUR prevents renal scarring
  C. Some patients with severe VUR have no renal scarring
  D. Male patients are more susceptible than female patients are
4. A 12-year-old girl is on a maximum dose of a calcium channel blocker (CCB) and hydrochlorothiazide for hypertension secondary to left reflux nephropathy. The most recent VCUG showed grade II VUR on the left side. A DMSA renal scan showed a differential GFR of 12% in the left kidney and 90% in the right kidney. An echocardiogram 2 weeks ago revealed moderate left ventricular hypertrophy. The patient’s compliance with the medication is questionable. Of the following, which ONE would be the most appropriate management for this patient?
  A. Deflux procedure
  B. Emphasize the need for compliance with medications
  C. Left nephrectomy
  D. Change CCB to an angiotensin-converting enzyme inhibitor
